# Selective receptor expression restricts Nipah virus infection of endothelial cells

**DOI:** 10.1186/1743-422X-5-142

**Published:** 2008-11-26

**Authors:** Stephanie Erbar, Sandra Diederich, Andrea Maisner

**Affiliations:** 1 Institute of Virology, Philipps University of Marburg, Marburg, Germany

## Abstract

Nipah virus (NiV) is a highly pathogenic paramyxovirus that causes severe diseases in animals and humans. Endothelial cell (EC) infection is an established hallmark of NiV infection *in vivo*. Despite systemic virus spread via the vascular system, EC in brain and lung are preferentially infected whereas EC in other organs are less affected. As *in vivo*, we found differences in the infection of EC in cell culture. Only brain-derived primary or immortalized EC were found to be permissive to NiV infection. Using a replication-independent fusion assay, we could show that the lack of infection in non-brain EC was due to a lack of receptor expression. The NiV entry receptors ephrinB2 (EB2) or ephrinB3 were only expressed in brain endothelia. The finding that EB2 expression in previously non-permissive aortic EC rendered the cells permissive to infection then demonstrated that EB2 is not only necessary but also sufficient to allow the establishment of a productive NiV infection. This strongly suggests that limitations in receptor expression restrict virus entry in certain EC subsets *in vivo*, and are thus responsible for the differences in EC tropism observed in human and animal NiV infections.

## Findings

Nipah virus (NiV) was identified in 1999 after an outbreak of fatal encephalitis among pig farmers in Malaysia [1]. Fruit bats of the genus *Pteropus *were identified as natural reservoir [2]. Together with the closely related Hendra virus, NiV represents the genus Henipavirus within the paramyxovirus family [3]. In contrast to most paramyxoviruses, henipaviruses cause diseases in many mammalian species including pigs, cats, horses, hamsters, guinea pigs and humans [4-7], and are classified as biosafety level 4 (BSL-4) pathogens.

Histopathological studies of NiV infections revealed that vascular endothelial cells (EC) are the predominant target cells of NiV [4,5,8,9]. Clinical disease, however, was affected by further tropism to non-vascular tissues (e.g. neurons in the brain). In humans, a widespread vasculitis is observed and NiV infection and syncytia formation is believed to trigger thrombosis and necrosis in the involved vessels. However, the extent of EC destruction due to NiV infection varies in different organs, and was found to be most prominent in small vessels in the central nervous system (CNS), the lung and the spleen, whereas other organs are less or not at all affected (liver) [1]. The capacity of EC in different organs to support virus replication is thus an important determinant for the clinical outcome of NiV infection. Aim of this study was to elucidate which cellular factor(s) determine what kind of EC can be productively infected. Properties of EC from different organs are known to be heterogenous [10], and several cell- or organ-type specific host components are described to either enhance or to interfere with different steps of viral replication such as surface-expressed C-type lectins (DC-SIGNR, LSECtin) which can promote virus attachment prior to receptor binding [11,12], or intracellular factors influencing uncoating, viral RNA replication, viral protein synthesis or virus assembly [13-16]. Besides these host cell factors, major candidates deciding on cell tropism are specific viral receptors. In the case of NiV, the main entry receptor is ephrinB2 (EB2) [17,18], a transmembrane protein which is highly conserved among all mammalian species. EB2 is a ligand of EphB4 receptors and is involved in neurogenesis and angiogenesis [19-22]. In the vasculature, EB2 is selectively expressed on arterial EC to fulfill its function in angiogenesis and neovascularization [23]. Even if EB2 is generally expressed in arteries and arterioles, the expression levels vary greatly in different organs. Highest levels of EB2 expression were reported in lung and colon, EB2 expression in brain tissue was only middle and EB2 mRNA levels detected in spleen and liver were low [24]. Since this *in vivo *expression profile does not correlate with the NiV organ tropismus, it remains to be determined if differences in organ-specific host factors other than receptor expression are responsible for the observed differences in EC infection.

**Figure 1:**
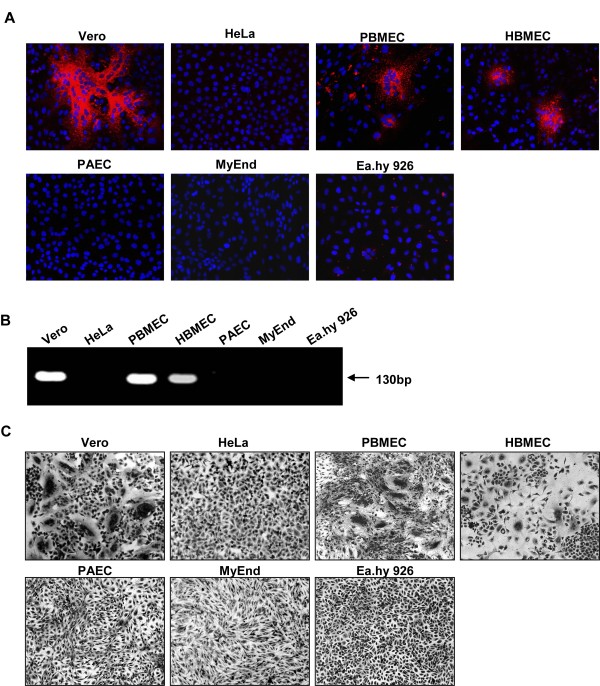
**NiV infection and NiV glycoprotein-mediated cell-to-cell fusion in different model EC**. PBMEC, HBMEC, PAEC, MyEnd, Ea.hy 926 and control Vero and HeLa cells were infected with NiV at a MOI of 0.2. (A) At 24 h p.i., cells were fixed, incubated with a NiV-specific guinea pig antiserum and visualized with rhodamine-conjugated secondary antibodies. Nuclei were counterstained with DAPI. (B) At 48 h p.i., viral RNA was isolated from supernatants of infected cells. RT-PCR was performed using NP-specific primers (NPfor binds at bp 1160–1179 and NPrev binds at bp 1271–1292). (C) HeLa cells were cotransfected with plasmids encoding the NiV glycoproteins F and G and were incubated at 33°C. 22 h after transfection, control cells and the different EC types were overlayed with NiV F- and G-expressing HeLa cells. 24 h later, cell-to-cell fusion was visualized by Giemsa staining. Magnification, ×20.

First, we assessed if the differences in EC infection reported for *in vivo *infection can also be observed in cell culture. For this we used the following model EC: PBMEC (primary porcine microvascular endothelial cells) freshly isolated from pig brain according to the protocol described in [25]; HBMEC (human brain endothelial cells [26]); PAEC (porcine aortic endothelial cells) [27]; MyEnd cells (mouse myocard endothelial cells) [28] and Ea.hy 926 cells derived from human umbilical vein endothelial cells [29]. As a control, Vero cells (permissive to NiV infection) and non-permissive HeLa cells were used [30]. For infection studies, cells were seeded on coverslips, grown to confluency and subsequently infected with NiV at a multiplicity of infection (MOI) of 0.2. All work with live virus was performed under BSL-4 conditions as described previously [31]. At 24 h post infection (p.i.), cells were fixed with 4% paraformaldehyde for 48 h. Virus-positive cells were detected after permeabilization using a NiV-specific guinea pig antiserum and rhodamine-conjugated secondary antibodies. As expected, large multinucleated syncytia were found in control Vero cells whereas HeLa cells were not infected. Among the tested model EC, only PBMEC and HBMEC allowed NiV replication. In both cell types, NiV-positive syncytia could be detected. All other EC types did not show any sign of infection. Supporting the finding that only PBMEC and HBMEC are permissive to NiV infection, viral RNA was detected by RT-PCR in the cell supernatants (Fig. 1B).

To determine if the lack of productive NiV infection in non-brain derived EC is due to an intracellular replication block, or is rather due to a defective receptor interaction preventing virus entry, we analyzed the ability of the different cells to support NiV glycoprotein mediated fusion by an overlay fusion assay. NiV G and F proteins coexpressed on the cell surface mediate cell-to-cell fusion with contacting receptor-positive cells; this assay therefore allows testing of cell lines for functional receptor expression independent of NiV replication. HeLa cells which do not support NiV-mediated fusion were transfected with plasmids encoding for the NiV glycoproteins F and G [32]. At 22 h post transfection (p.t.), F/G-expressing HeLa cells were detached by trypsin/EDTA treatment, and 1 × 10^5 ^cells were overlayed on EC monolayers grown on coverslips. 22 h later, cell-to-cell fusion was visualized by Giemsa staining. Fig. 1C clearly demonstrates that syncytia formation is only supported by Vero cells (positive control), as well as by PBMEC and HBMEC, the two brain-derived EC. Neither aortic, myocard nor umbilical cord EC (PAEC, MyEnd, Ea.hy 926) fused with NiV glycoprotein expressing HeLa cells suggesting that deficient replication in these cells is due to the lack of functional NiV receptors.

**Figure 2:**
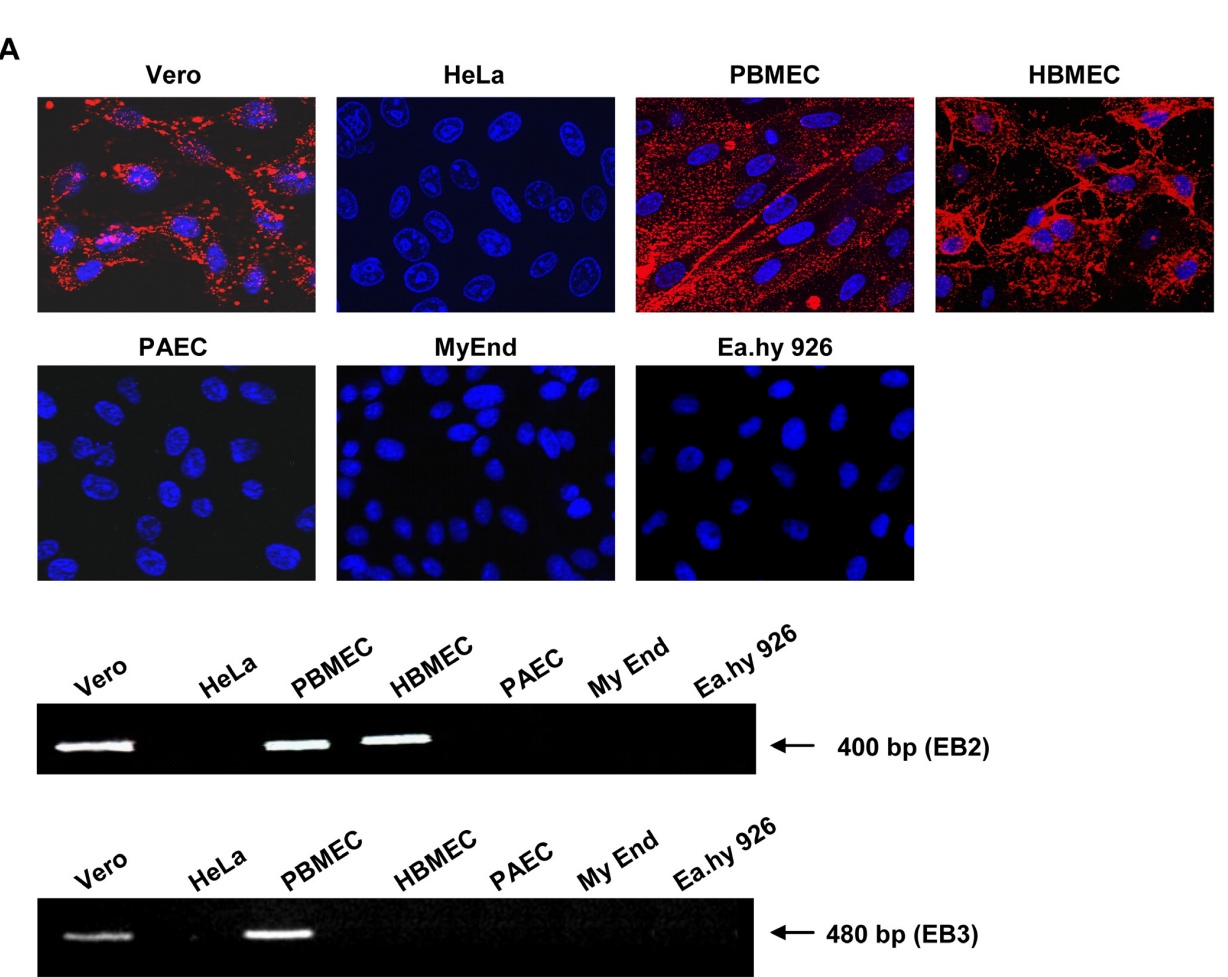
**EB2 and EB3 expression in model EC**. (A) EC were cultured on coverslips and immunostaining was performed using recombinant EphB4/Fc and rhodamine-conjugated secondary antibodies. Nuclei were visualized by DAPI staining. Magnification, ×100. (B) mRNA was extracted from 5 × 10^5 ^cells by standard procedures and subjected to RT-PCR with EB2-specific primers. (C) RT-PCR with EB3-specific primers.

To confirm that variations in receptor expression are responsible for the observed differences in EC infection, EB2 expression in the different cells was analyzed by surface immunostaining. For this, cells were fixed with 4% paraformaldehyde, incubated with a recombinant mouse EphB4/Fc, a soluble EB2 receptor fused to the Fc region of human IgG (R&D Systems) and rhodamine-coupled secondary antibodies [31]. As shown in Fig. 2A, EB2 staining was only found in Vero cells, HBMEC and PBMEC. To confirm the lack of EB2 expression in the cells non-permissive to NiV infection, RNA was isolated from 5 × 10^5 ^of each cell line using an RNeasy Mini kit (Qiagen). Subsequently, RT-PCR was performed using EB2-specific primers [33]. In agreement with the immunostaining, Fig. 2B clearly demonstrates that EB2 mRNA was only present in Vero cells and brain-derived EC.

It was reported that in addition to EB2, ephrinB3 (EB3) may serve as alternative receptor for NiV. *In vivo*, EB3 is expressed in the CNS and likely accounts for specific aspects of NiV pathology in the brain [34]. Even if EB3 is not involved in angiogenesis [35], and is thus not assumed to be expressed on EC *in vivo*, expression of EB3 in our model EC was analyzed by an EB3-specific RT-PCR [33]. Fig. 2C shows that besides Vero cells, only PBMEC express small amounts of EB3 mRNA. This revealed that EB3 is not only expressed in brain parenchyma but also in brain EC and might therefore be used as alternate receptor in this cell type in the absence of EB2. However, EB3 expression is most likely not involved in NiV binding and syncytia formation in our PBMEC, because these cells express high amounts of EB2, and it was shown that NiV-G binds to EB2 with much higher affinity [34].

**Figure 3:**
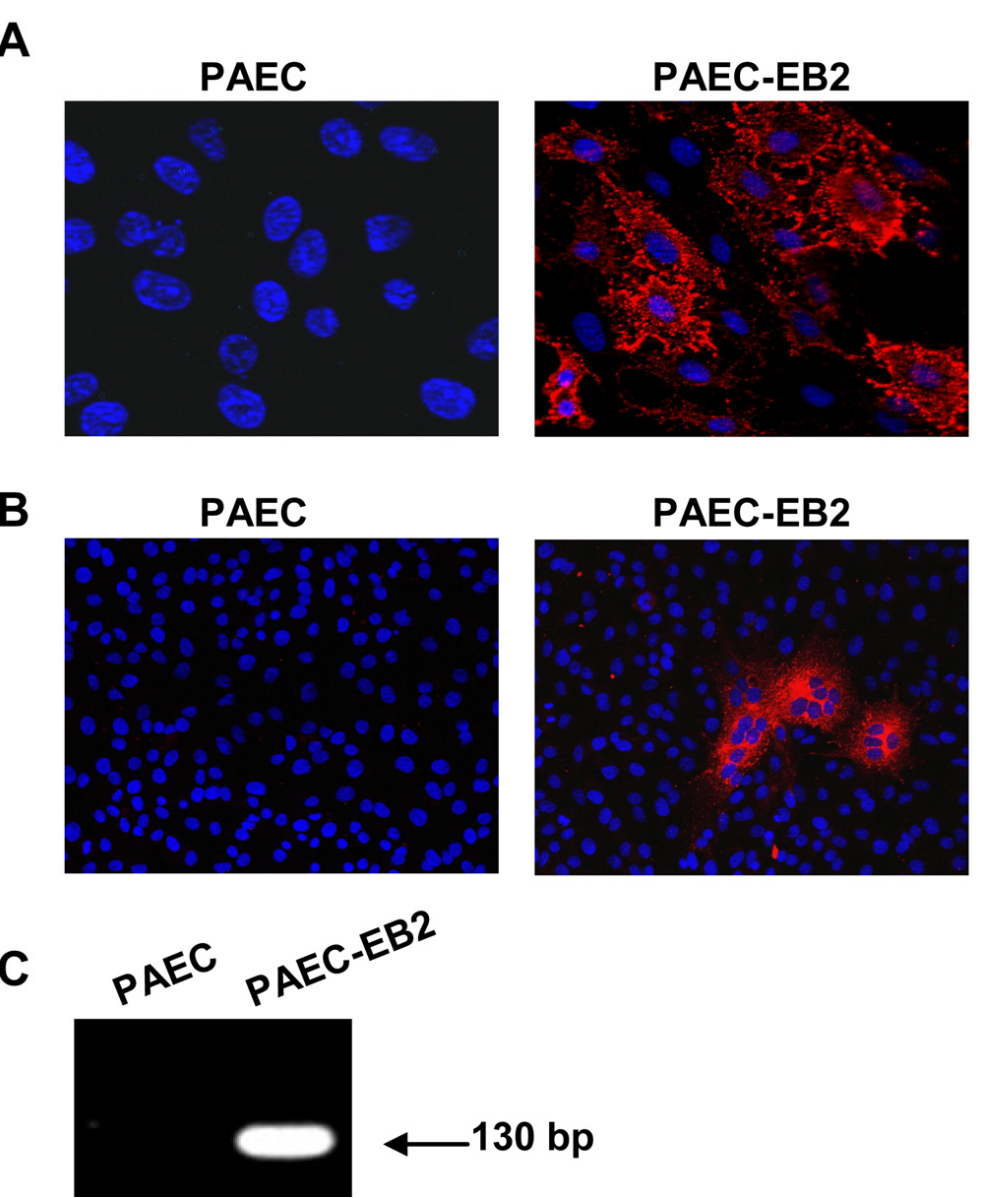
**NiV infection of EB2 expressing PAE cells (PAEC-EB2)**. (A) PAEC and PAEC-EB2 cells were immunostained for EB2 as described in the legend to Fig. 2B. Magnification, ×100. (B) PAEC and PAEC-EB2 were infected with NiV at a MOI of 0.2. At 24 h p.i., cells were fixed and immunostaining was performed as described in the legend to Fig. 1A. Magnification, ×20. (C) 48 h p.i., mRNA was isolated from the cell supernatant, and RT-PCR was performed with NP-specific primers.

To analyze if receptor expression is the only determinant responsible for selective infection of brain-derived EC, or if there are further brain-specific cellular factors responsible for this tropism, we analyzed NiV infection of aortic EC (PAEC) which had been stably transfected with the human EB2 gene (PAEC-EB2) [27]. As shown in Fig. 3A, EB2 is readily expressed on the surface of these cells. We then infected PAEC-EB2 with NiV (MOI of 0.2), visualized virus-positive cells by immunostaining at 24 h p.i. and analyzed virus release into the supernatant by RT-PCR at 48 h p.i.. In contrast to wildtype PAEC, PAEC-EB2 clearly supported productive NiV infection. NiV-positive syncytia were clearly detectable in PAEC-EB2 (Fig. 3B) and viral RNA was found in cell supernatants (Fig. 3C). This revealed that EB2 expression in non-permissive PAEC rendered these cells fully permissive to NiV infection, and thus indicates that EB2 expression is necessary and sufficient for NiV infection of EC. Together, our results clearly indicate a strict correlation of EB2 expression in EC and permissiveness to NiV infection in cell culture. Only brain-derived primary or immortalized EC were receptor-positive and supported NiV glycoprotein-mediated fusion as well as NiV infection. Even if additional host factors such as interferon-induced antiviral proteins [16,36] might influence NiV infection of EC in different organs *in vivo*, our data strongly suggest that variations in the receptor expression are the important key factor for EC tropism in the course of systemic NiV infections.

## Competing interests

The authors declare that they have no competing interests.

## Authors' contributions

SE carried out most of the experiments and helped to draft the manuscript. SD performed all work under BSL-4 conditions. AM designed the study, helped with analysis and the interpretation of the data and drafted the manuscript. All authors read and approved the final manuscript.
